# Longitudinal Associations Between Anxiety, Frailty, and Aging Perceptions Among Hispanic Older Adults

**DOI:** 10.1093/geroni/igaf122.2489

**Published:** 2025-12-31

**Authors:** Janet Lopez, Dahee Kim, Yi Liu, Rui Xie, Abigail Tice, Michael Dino, Ayse Malatyali, Ladda Thiamwong

**Affiliations:** University of Central Florida, Orlando, Florida, United States; University of Central Florida, Orlando, Florida, United States; University of Central Florida, Orlando, Florida, United States; University of Central Florida, Orlando, Florida, United States; University of Central Florida, Orlando, Florida, United States; University of Central Florida, University of Central Florida, Florida, United States; University of Central Florida, Orlando, Florida, United States; University of Central Florida, Orlando, Florida, United States

## Abstract

Aging perceptions significantly impact health outcomes for older adults, especially among Hispanic populations facing unique challenges. While cross-sectional studies have examined relationships between psychological factors, physical health indicators, and aging perceptions, longitudinal relationships remain understudied, particularly among Hispanic older adults. This study aimed to examine how anxiety and frailty are associated with aging perceptions over time. We analyzed data from 87 Hispanic older adults who participated in a Technology-Based Body and Mind Intervention to Prevent Falls. They were recruited from independent living facilities and community centers in Central Florida. Generalized Linear Mixed Models (GLMMs) examined relationships between aging perceptions (B-APQ total score and five subscales: timeline-chronic, emotion representation, control-positive, consequence-positive, control consequence-negative) and key predictors, including anxiety (GAI-SF) and frailty (short FRAIL scale) across four time points. Models incorporated these predictors as fixed effects with subject-level random effects to account for repeated measures. Outcomes showed that anxiety was significantly associated with higher scores on the B-APQ Chronic subscale (β = 0.358, SE = 0.138, p = 0.010). Both anxiety (β = 0.362, SE = 0.134, p = 0.007) and frailty (β = 0.546, SE = 0.266, p = 0.041) were significantly associated with increased emotional representation scores. No significant associations were found for the remaining B-APQ subscales. Our study highlights the association between psychological factors and frailty, emphasizing the need for culturally sensitive interventions to promote healthy aging. Its findings underscore the importance of addressing anxiety and frailty issues to improve aging perceptions and enhance well-being among Hispanic older adults. This approach has the potential to positively impact aging perceptions in this population, leading to improved health outcomes.

